# Influence of Experimental End Point on the Therapeutic Efficacy of Essential and Additional Antidotes in Organophosphorus Nerve Agent-Intoxicated Mice

**DOI:** 10.3390/toxics10040192

**Published:** 2022-04-15

**Authors:** Jiri Kassa, Christopher M. Timperley, Mike Bird, A. Christopher Green, John E. H. Tattersall

**Affiliations:** 1 Department of Toxicology and Military Pharmacy, Faculty of Military Health Sciences, University of Defence, 60200 Brno, Czech Republic; 2 Chemical, Biological and Radiological Division, Defence Science and Technology Laboratory (DSTL), Salisbury SP4 0JQ, UK; cmtimperley@mail.dstl.gov.uk (C.M.T.); mbird@dstl.gov.uk (M.B.); acgreen@dstl.gov.uk (A.C.G.); jtattersall@dstl.gov.uk (J.E.H.T.)

**Keywords:** atropine, MB327, nerve agents, oximes, mice

## Abstract

The therapeutic efficacy of treatments for acute intoxication with highly toxic organophosphorus compounds, called nerve agents, usually involves determination of LD_50_ values 24 h after nerve agent challenge without and with a single administration of the treatment. Herein, the LD_50_ values of four nerve agents (sarin, soman, tabun and cyclosarin) for non-treated and treated intoxication were investigated in mice for experimental end points of 6 and 24 h. The LD_50_ values of the nerve agents were evaluated by probit-logarithmical analysis of deaths within 6 and 24 h of i.m. challenge of the nerve agent at five different doses, using six mice per dose. The efficiency of atropine alone or atropine in combination with an oxime was practically the same at 6 and 24 h. The therapeutic efficacy of the higher dose of the antinicotinic compound MB327 was slightly higher at the 6 h end point compared to the 24 h end point for soman and tabun intoxication. A higher dose of MB327 increased the therapeutic efficacy of atropine alone for sarin, soman and tabun intoxication, and that of the standard antidotal treatment (atropine and oxime) for sarin and tabun intoxication. The therapeutic efficacy of MB327 was lower than the oxime-based antidotal treatment. To compare the 6 and 24 h end points, the influence of the experimental end point was not observed, with the exception of the higher dose of MB327. In addition, only a negligible beneficial impact of the compound MB327 was observed. Nevertheless, antinicotinics may offer an additional avenue for countering poisoning by nerve agents that are difficult to treat, and synthetic and biological studies towards the development of such novel drugs based on the core bispyridinium structure or other molecular scaffolds should continue.

## 1. Introduction

Organophosphorus nerve agents are highly toxic chemical warfare agents. Their signs and symptoms can appear rapidly [1]. They inhibit acetylcholinesterase (AChE, EC 3.1.1.7) irreversibly [2]. Acetylcholine (ACh) then accumulates in the nervous system. The ACh overstimulates muscarinic (mAChRs) and nicotinic acetylcholine receptors (nAChRs) causing muscarinic and nicotinic effects: muscle twitching, lachrymation, miosis, salivation, seizures and coma [3]. Death arises from the inability to breathe, bronchial spasm and hypersecretion, and paralysis of the diaphragm and the brain respiratory centers [4,5].

The acute poisoning is usually treated by injecting a reactivator of the inhibited AChE (such as pralidoxime, HI-6, obidoxime or trimedoxime), an antimuscarinic (usually atropine to counter the muscarinic effects) and an anticonvulsant (e.g., diazepam to counter the centrally-mediated seizures) [1,2,3,4,5]. The treatment does not include drugs able to directly reduce the nicotinic effects. Its effectiveness is generally insufficiently broad because of the limitation of a single oxime to reactivate AChE inhibited by all nerve agents equally [6,7,8].

The search for more efficient therapies continues [9,10,11]. One option is to use an nAChR antagonist to counter the nicotinic effects [12]. Nicotinic antagonists administered with the aforementioned standard drug combination can help [13] as atropine cannot reverse the nerve agent-induced overstimulation of nAChRs [14]. Some bispyridinium compounds can do this, by blocking the nAChR open ion channel and/or orthosteric sites, or by positive allosteric modulation of nAChRs [15,16,17,18,19,20].

The bispyridinium non-oxime [1,1′-(propane-1,3-diyl) bis(4-tert-butyl pyridinium) diiodide] (MB327) was prepared at Dstl Porton Down, Salisbury, United Kingdom, and was found to be effective for treating the actions of nerve agents in guinea pigs [21,22]. MB327 has been shown to have a positive therapeutic effect due to a non-competitive action at muscle type nAChRs and positive allosteric activity at neuronal nAChRs [20].

MB327 is more effective over shorter timescales due its rapid elimination from the circulation (in guinea pigs) [23]. The therapeutic efficacy of antidotes is most often investigated 24 h after the nerve agent challenge. However, given that clearance of the treatment drugs, including MB327 and atropine, may impact their efficacy, the use of an earlier end point may provide a more realistic or useful assessment of their benefit as immediate or first aid treatments.

**Figure 1:**
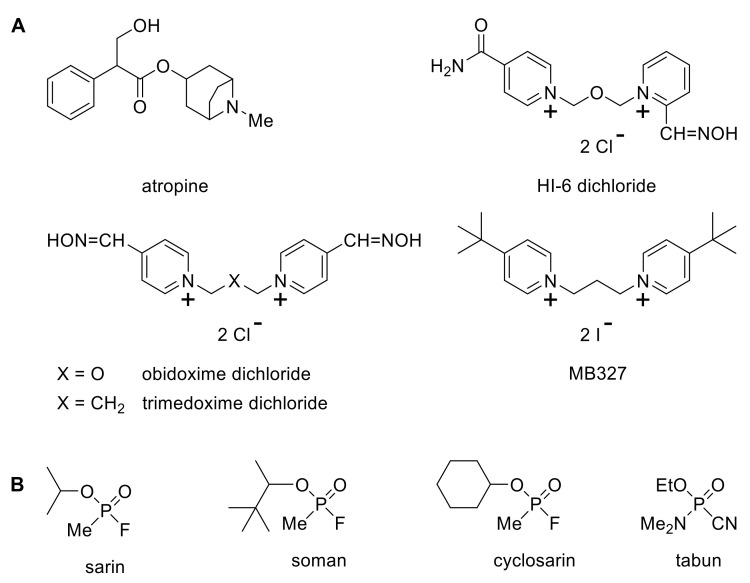
Components (**A**) of the therapies tested—the antimuscarinic atropine, the oximes HI-6, obidoxime and trimedoxime, and the antinicotinic MB327—against the panel of nerve agents shown (**B**).

The present study measured the 6 h LD_50_ of sarin, soman, tabun or cyclosarin in mice either alone, or in the presence of atropine with or without an effective oxime (HI-6, obidoxime or trimedoxime) and/or MB327 (Figure 1). The results were compared to the 24 h LD_50_ measurements.

## 2. Materials and Methods

### 2.1. Animals

Male NMRI mice aged 7 weeks, weighing 30–35 g from VELAZ (Prague, Czech Republic) were housed in polypropylene solid bottom cages in climate- and access-controlled rooms (22 ± 2 °C and 50 ± 10% relative humidity) with light from 07:00 to 19:00 h and access to standard food and tap water ad libitum. The mice were acclimatized in the laboratory vivarium for 14 days before the experiments commenced. They were divided into groups of 6 animals and handled under the supervision of the Ethics Committee of the Faculty of Military Health Sciences in Hradec Kralove (Czech Republic).

### 2.2. Chemicals

The nerve agents were obtained in 85–90% purity (by acidimetric titration) from the Military Technical Institute in Brno (Czech Republic). Stock solutions of these (1 mg/mL) were prepared in propylene glycol 3 days before starting the experiments. The solutions were diluted with saline immediately before administration. HI-6, obidoxime and trimedoxime dichlorides were synthesized in the Department of Toxicology and Military Pharmacy of the Faculty of Military Health Sciences in Hradec Kralove. Their purity was assessed by high performance liquid chromatography (HPLC) with ultraviolet detection (310 nm) and exceeded 95% [24]. MB327 of 99% purity was synthesized at Dstl Porton Down [22]. Other chemicals of a similar analytical grade were purchased commercially and used as received. All substances were administered by intramuscular (i.m.) injection at a volume of 10 mL/kg body weight (b.w.). The volume of administered substances was higher than recommended for i.m. administration in mice but there were no adverse effects observed.

### 2.3. In Vivo Experiments

The LD_50_ values of the nerve agents and 95% confidence limits in mice were evaluated by probit-logarithmical analysis of deaths within 6 and 24 h of i.m. administration of the nerve agent at 5 different doses, using 6 mice per dose [25]. The intoxicated mice were treated i.m. with atropine or with an oxime and/or MB327. An effective oxime was used for each nerve agent: obidoxime for sarin, trimedoxime for tabun, and HI-6 for soman and cyclosarin [4,7,26,27].

The oximes were delivered at equitoxic doses (obidoxime 5.8 mg/kg; trimedoxime 5.3 mg/kg; and HI-6 at 25 mg/kg) corresponding to 5% of their respective LD_50_. MB327 was administered at doses (16.7 and 33.4 mg/kg) corresponding to 10 and 20% of its respective LD_50_ [22]. Equitoxic doses were used to ensure that sufficiently safe doses with respect to their different toxicities were given. Consistent with previous work, atropine (10 mg/kg) was injected at a dose approximating 2% of its LD_50_ [28]. All antidotes were applied as an admix 1 min after i.m. administration of the nerve agent.

The LD_50_ values of the nerve agents and 95% confidence limits in mice treated with the antidotes were evaluated by the method used to determine the toxicity of the nerve agents without antidotal treatment [25]. The therapeutic efficacy of the antidotal combinations was expressed as a protective ratio A (LD_50_ value of the nerve agent in protected mice/LD_50_ value of the nerve agent in unprotected mice) for the 6 or 24 h end points. The influence of end point on the therapeutic efficacy of the antidotes was expressed as a protective ratio B (LD_50_ value of the nerve agent in mice for the 6 h end point/LD_50_ value of the nerve agent in mice for the 24 h end point). Statistical significance was determined by a one-way ANOVA test with Tukey’s post hoc test and differences were considered significant when *p* < 0.05 [25].

## 3. Results

Clinical signs in mice intoxicated with sarin, soman, tabun and cyclosarin included salivation, lachrymation, nasal secretion and tonic-clonic convulsions. Signs were observed within a few minutes of administration of the nerve agents. In the case of treatment for nerve agent poisoning, nerve agent-induced signs and symptoms did not disappear but their intensity was decreased regardless of the type of nerve agent used. The decrease in the intensity of toxic signs and symptoms was especially observed when atropine in combination with an oxime was used as a treatment of nerve agent poisoning. Observations and potencies of the antidotes studied are now discussed in turn.

### 3.1. The Efficacy of Antidotes (Atropine, Obidoxime, MB327) for Prevention of Sarin Lethality

The dependence of the therapeutic efficacy of the various combinations of treatments on the selected experimental end point is summarized in Table 1. The times to death were similar for all treatment groups. Most mice died within the first hour. The average time to death within the first 6 h was 7.4 min for non-treated animals and 8.1 min for treated animals. Only in the atropine alone group did some deaths occur after 6 h. All deaths occurred within 6 h of intoxication for the other groups: atropine with obidoxime or MB327, and atropine with obidoxime and MB327. Atropine increased the LD_50_ of sarin 1.25-fold at 6 h and 1.10-fold at 24 h. The combination of atropine and obidoxime increased it 1.45-fold. With the lower dose of MB327, it increased it 1.21-fold, and with the higher dose of MB327 it increased it 1.23-fold. Atropine and obidoxime and the higher dose of MB327 increased it 1.80-fold, regardless of the end point. The efficacy of atropine and obidoxime was higher than that of atropine and MB327 regardless of the dose of the latter. With atropine and MB327, a slight increase in protection ratio versus atropine alone was seen at 24 h but the difference was not significant. A significant benefit of MB327 on the efficacy of atropine and obidoxime was not observed.

### 3.2. The Efficacy of Antidotes (Atropine, HI-6, MB327) for Prevention of Soman Lethality

The data for soman appear in Table 2. The times to death differed slightly between the treatment groups; nevertheless, most mice died within the first hour. The average time to death within the first 6 h was 6.7 min for non-treated animals and 22.4 min for treated animals. Only for atropine with the higher dose of MB327 did deaths occur after 6 h. Thus, with the higher dose of MB327 the LD_50_ of soman was increased 1.48-fold at 6 h, but only 1.31-fold at 24 h. In the other groups—atropine alone, or with HI-6 or the lower dose of MB327, or with HI-6 and the higher dose of MB327—all deaths occurred within 6 h. Atropine increased the LD_50_ 1.36-fold, atropine and HI-6 increased it 1.81-fold, atropine and the lower dose of MB327 increased it 1.32-fold, and atropine and HI-6 with a higher dose of MB327 increased it 1.80-fold, at both end points. The efficacy of HI-6 with atropine exceeded that of atropine and MB327 regardless of the MB327 dose and end point. With atropine and the higher dose of MB327, a small increase in protection ratio compared to atropine alone was apparent at 6 h but the difference was not significant. A significant benefit of MB327 on the efficacy of atropine and HI-6 was not observed.

### 3.3. The Efficaccy of Antidotes (Atropine, Trimedoxime, MB327) for Prevention of Tabun Lethality

The tabun results feature in Table 3. Times to death differed slightly between treatment groups. All deaths occurred within 6 h in mice given atropine and trimedoxime, atropine and the lower dose of MB327, and atropine with trimedoxime and MB327. Most mice died within the first hour. The average time to death within the first 6 h was 14.3 min for non-treated animals and 17.6 min for treated animals. With atropine alone, and with atropine in combination with the higher dose of MB327, some deaths occurred after 6 h. Thus, atropine increased the LD_50_ of tabun 1.20-fold at 6 h but only 1.09-fold at 24 h. Atropine and the higher dose of MB327 increased it 1.52-fold at 6 h and only 1.32-fold at 24 h. Atropine and the lower dose of MB327 increased it only 1.20-fold, atropine and trimedoxime increased it 1.95-fold, and atropine and trimedoxime and the higher dose of MB327 increased it 2.06-fold. With atropine and the higher dose of MB327, the protection ratio increased compared to atropine alone, at both experimental end points but the differences were not significant. The efficacy of atropine and trimedoxime was higher than that of atropine and MB327 regardless of the dose of the latter and end point. A significant benefit of MB327 on the efficacy of atropine and trimedoxime was not observed.

### 3.4. The Efficacy of Antidotes (Atropine, HI-6, MB327) for Prevention of Cyclosarin Lethality

The efficacies of the antidotes to cyclosarin appear in Table 4. A difference in distribution of times to death among treatment groups was not observed; all deaths occurred within the first 6 h. Most mice died within the first hour. The average time to death within the first 6 h was 9.3 min for non-treated animals and 12.5 min for treated animals. With atropine alone the LD_50_ of cyclosarin increased 1.31-fold. In combination with HI-6 it increased by 3.15-fold, with the lower or higher dose of MB327 it increased it 1.24- or 1.34-fold, respectively, and with HI-6 and the higher dose of MB327 it increased it 3.12-fold. Atropine and the higher dose of MB327 produced a slightly increased therapeutic efficacy compared to atropine with the lower dose of MB327 but the difference was not significant. The efficacy of atropine and HI-6 was superior to that of atropine and MB327. A benefit of MB327 on the efficacy of atropine and HI-6 was not observed.

## 4. Discussion

Oximes are key components of nerve agent antidotes due to their capability to reactivate nerve agent-inhibited AChE. However, their therapeutic efficacy is restricted, as reactivation of inhibited AChE is not universally effective because all nerve agents and current oximes are generally ineffective in the central compartment due to their difficulty crossing the blood–brain barrier [4]. Generally, the reactivating efficacy of each oxime depends on the chemical structure of the nerve agent and it is not the same for all nerve agents studied. Therefore, one of the most effective oximes was chosen for each nerve agent (obidoxime for sarin, trimedoxime for tabun, and HI-6 for soman and cyclosarin [4,7,26,27]). Although obidoxime, used for the treatment of sarin poisoning, has a lower efficacy against sarin than the oxime HI-6 [26], it was used for sarin poisoning in this study due to the need to compare our results with previously published data where obidoxime was used for the treatment of sarin poisoning.

Additionally, anticholinergic drugs such as atropine cannot counter the overstimulation of nAChRs. Therefore, novel potential antidotes, based on bispyridinium non-oxime structures, were developed to mitigate the overstimulation of nAChRs and to reduce the therapeutic reliance on oxime-based reactivation of AChE in the treatment of nerve agent poisoning [12,29].

Several bispyridinium compounds have been shown to counter the acute toxicity of nerve agents [12,21,22,23,30]. Their efficacy hinges on modulating nAChRs at the ion channel, orthosteric ACh binding sites, or both [31,32]. One of the most promising is MB327. It has been shown to have a positive therapeutic effect due to a non-competitive antagonistic action at muscle type nAChRs and has been found to have a positive allosteric activity at muscle and neuronal AChRs [20,33,34]. MB227 has been characterized as a re-sensitizer of the desensitized nAChR. MB327 is thereby capable of restoring its functional activity and restoring nerve agent-induced irreversibly disrupted neurotransmission at the neuromuscular junctions. The re-sensitizing effect of MB327 is probably based on its positive allosteric activity, as was repeatedly demonstrated [32,35]. Two putative binding sites were found for MB327. Both sites are located inside the channel: one in the extracellular domain between the γ and α subunits, and the other between β and δ subunits in the transmembrane region [17,33,34]. By comparison, MB327 does not interact with the orthosteric ACh binding sites [16]. It is known that conventional nAChR antagonists have a low therapeutic index between sufficient antagonism and muscle paralysis and, therefore, they have a negative impact on respiration However, MB327, which acts as a re-sensitizer of the desensitized nAChR, has a low impact on respiration when administered at recommended doses [13]. In addition, MB327, like other bispyridinium non-oxime compounds, is a charged polar species. It has a high aqueous solubility and comparatively low hydrophobicity. It is therefore expected to exert its biological action mainly in the periphery rather than in the brain, and to have a low capacity to cross the blood–brain barrier.

In the present study, the significant benefit of MB327 for the therapeutic efficacy of standard antidotal treatment of nerve agent poisoning was not shown.

To determine the optimal potential of antidotes, a pharmacologically relevant time following intoxication should be selected based on the pharmacokinetics and mechanisms of action of the drugs tested. An effective concentration of MB327 in the blood and at key nicotinic synapses in tissues is required for nicotinic antagonism and consequent protection. Due to the predicted rapid elimination of MB327 from the body [23], a decrease in the efficacy of MB327 from 6 to 24 h was anticipated. However, a slight reduction in the efficacy of MB327 from 6 to 24 h was only observed for the higher dose of MB327 for soman and tabun intoxication.

To compare the influence of the end point on the therapeutic efficacy of MB327 with other MB antinicotinics, the analogue MB408 showed a higher decrease in its efficacy from 6 to 24 h in the case of intoxication with nerve agents, especially sarin and soman [36]. Generally, the changes in protection ratio seen with atropine with MB327 at 6 h compared to 24 h were small, were only observed for the higher MB327 dose, and were not statistically significant. In contrast, the effects of MB327 in guinea pigs showed a very marked increase in protection at a 6 h compared to a 24 h end point [37]. This disparity may reflect MB327 pharmacokinetic differences between the mouse and guinea pig: in size, absorption, distribution, metabolism and elimination.

The present and previous studies in mice [36] and in guinea pigs [37] demonstrate that earlier experimental end points can show that certain treatments can provide increased protection against acute intoxication by nerve agents. This is also supported theoretically by the reversible, pharmacological mechanism of action of certain antidotes, and therefore a reliance on their pharmacokinetics, ensuring that therapeutically effective drug concentrations are maintained. Importantly, then, the shorter experimental end point may reduce the risk of falsely rejecting effective treatments, particularly if these are intended for immediate, first-aid treatment of nerve agent casualties. Our study has limitations because only one animal species and one end point (besides the standard 24 h) were used. Due to species differences, especially in the pharmacokinetics of MB327 (and analogues), the end point chosen should be matched to the animal species studied, as different species or end points may provide different results.

## 5. Conclusions

Although MB327 may provide limited benefit for the treatment of nerve agent poisoning in the absence of an oxime, the data from the evaluation of the therapeutic efficacy of MB327 in this particular study do not support the presumption that this non-competitive antinicotinic may significantly benefit the standard antidotal treatment (atropine + oxime) of nerve agent intoxication, at least in mice. Its effectiveness did not approach any of the oximes studied in this animal species. Therefore, atropine and an oxime should continue to be the core elements of treatment. As only a negligible beneficial impact of the compound MB327 was observed, novel compounds with similar action to MB327 but which exhibit better nicotinic selectivity and increased antinicotinic efficacy should be developed.

## Figures and Tables

**Table 1:** The effect of experimental endpoint on the protection provided against lethal effects of sarin in mice.

Treatment	LD_50_ (μg/kg) ± 95% Confidence Limit—6 h	Protective Ratio A	LD_50_ (μg/kg) ± 95% Confidence Limit—24 h	Protective Ratio A	Protective Ratio B
-----	328.2 (270.1–360.2)	----	328.2 (270.1–360.2)	----	----
Atropine	411.9 (329.9–514.2)	1.25	361.2 (324.9–430.7)	1.10	1.14
Atropine + obidoxime	476.9 (375.8–539.1) *	**1.45**	476.9 (375.8–539.1) *	**1.45**	**1.00**
Atropine + MB327 (lower dose)	397.8 (354.1–446.8)	1.21	397.8 (354.1–446.8)	1.21	1.00
Atropine + MB327 (higher dose)	402.7 (269.1–496.3)	1.23	402.7 (269.1–496.3)	1.23	1.00
Atropine + obidoxime + MB327	591.6 (456.9–766.1) *	**1.80**	591.6 (456.9–766.1) *	**1.80**	**1.00**

* *p* < 0.05 (comparison with the untreated values). Bold values are significantly different from untreated values at the level of *p* < 0.05.

**Table 2:** The effect of experimental endpoint on the protection provided against the lethal effects of soman in mice.

Treatment	LD_50_ (μg/kg) ± 95% Confidence Limit—6 h	Protective Ratio A	LD_50_ (μg/kg) ± 95% Confidence Limit—24 h	Protective Ratio A	Protective Ratio B
-----	110.5 (79.4–137.1)	----	110.5 (79.4–137.1)	----	----
Atropine	150.1 (92.8–190.1)	1.36	150.1 (92.8–190.1)	1.36	1.00
Atropine + HI-6	199.9 (140.6–282.3) *	**1.81**	199.9 (140.6–282.3) *	**1.81**	**1.00**
Atropine + MB327 (lower dose)	145.7 (124.7–186.0)	1.32	145.7 (124.7–186.0)	1.32	1.00
Atropine + MB327 (higher dose)	163.1 (137.6–225.5) *	**1.48**	144.4 (117.9–196.8)	1.31	1.13
Atropine + HI-6 + MB327	198.8 (158.4–248.3) *	**1.80**	198.8 (158.4–248.3) *	**1.80**	**1.00**

* *p* < 0.05 (comparison with the untreated values). Bold values are significantly different from untreated values at the level of *p* < 0.05.

**Table 3:** The effect of experimental endpoint on the protection provided against the lethal effects of tabun in mice.

Treatment	LD_50_ (μg/kg) ± 95% Confidence Limit—6 h	Protective Ratio A	LD_50_ (μg/kg) ± 95% Confidence Limit—24 h	Protective Ratio A	Protective Ratio B
-----	420.5 (370.8–470.2)	----	420.5 (370.8–470.2)	----	----
Atropine	504.2 (447.4–533.3)	1.20	458.3 (405.2–494.0)	1.09	1.10
Atropine + trimedoxime	818.9 (774.2–866.2) *^,x^	**1.95**	818.9 (774.2–866.2) *^,x^	**1.95**	**1.00**
Atropine + MB327 (lower dose)	503.7 (466.5–543.9)	1.20	503.7 (466.5–543.9)	1.20	1.00
Atropine + MB327 (higher dose)	639.4 (492.2–822.3) *	**1.52**	554.2 (472.7–602.9) *	**1.32**	**1.15**
Atropine + trimedoxime + MB327	866.0 (738.9–1017.5) *^,x^	**2.06**	866.0 (738.9–1017.5) *^,x^	**2.06**	**1.00**

* *p* < 0.05 (comparison with the untreated values). **^x^** *p* < 0.05 (comparison with the values treated with atropine alone). Bold values are significantly different from untreated values and values treated with atropine alone at the level of *p* < 0.05.

**Table 4:** The effect of experimental endpoint on the protection provided against the lethal effects of cyclosarin in mice.

Treatment	LD_50_ (μg/kg) ± 95% Confidence Limit—6 h	Protective Ratio A	LD_50_ (μg/kg) ± 95% Confidence Limit—24 h	Protective Ratio A	Protective Ratio B
-----	248.4 (192.8–287.9)	----	248.4 (192.8–287.9)	----	----
Atropine	326.8 (254.9–417.7)	1.31	326.8 (254.9–417.7)	1.31	1.00
Atropine + HI-6	782.9 (680.6–856.4) *^,x^	**3.15**	782.9 (680.6–856.4) *^,x^	**3.15**	**1.00**
Atropine + MB327 (lower dose)	309.1 (268.0–394.4)	1.24	309.1 (268.0–394.4)	1.24	1.00
Atropine + MB327 (higher dose)	333.4 (247.1–400.9)	1.34	333.4 (247.1–400.9)	1.34	1.00
Atropine + HI-6 + MB327	775.9 (635.3–878.8) *^,x^	**3.12**	775.9 (635.3–878.8) *^,x^	**3.12**	**1.00**

* *p* < 0.05 (comparison with the untreated values). **^x^** *p* < 0.05 (comparison with the values treated with atropine alone). Bold values are significantly different from untreated values and values treated with atropine alone at the level of *p* < 0.05.

## Data Availability

The data from this study are available on request from the corresponding author. They are not publicly available due to ethical restrictions.
